# Single-cell expression noise and gene-body methylation in *Arabidopsis thaliana*

**DOI:** 10.1038/s41437-018-0181-z

**Published:** 2019-01-16

**Authors:** Robert Horvath, Benjamin Laenen, Shohei Takuno, Tanja Slotte

**Affiliations:** 1 Department of Ecology, Environment and Plant Sciences, Science for Life laboratory, Stockholm University, Stockholm, 106 91 Sweden; 2 Department of Evolutionary Studies of Biosystems, SOKENDAI (The Graduate University for Advanced Studies), Hayama, Kanagawa Japan

**Keywords:** DNA methylation, Gene expression

## Abstract

Gene-body methylation (gbM) refers to an increased level of methylated cytosines specifically in a CG sequence context within genes. gbM is found in plant genes with intermediate expression level, which evolve slowly, and is often broadly conserved across millions of years of evolution. Intriguingly however, some plants lack gbM, and thus it remains unclear whether gbM has a function. In animals, there is support for a role of gbM in reducing erroneous transcription and transcription noise, but so far most studies in plants have tested for an effect of gbM on expression level, not noise. Here, we therefore tested whether gbM was associated with reduced expression noise in *Arabidopsis thaliana*, using single-cell transcriptome sequencing data from root quiescent centre cells. We find that gbM genes have lower expression noise levels than unmethylated genes. However, an analysis of covariance revealed that, if other genomic features are taken into account, this association disappears. Nonetheless, gbM genes were more consistently expressed across single-cell samples, supporting previous inference that gbM genes are constitutively expressed. Finally, we observed that fewer RNAseq reads map to introns of gbM genes than to introns of unmethylated genes, which indicates that gbM might be involved in reducing erroneous transcription by reducing intron retention.

## Introduction

In angiosperms, three different classes of cytosine methylation can be distinguished based on their sequence context: methylation in a CG (mCG) context, methylation in a CHG (mCHG) context and methylation in a CHH (mCHH) context, where H stands for A, C or T (Cokus et al. 2008; Lister et al. 2008; Niederhuth and Schmitz 2014; Niederhuth et al. 2016; Bewick et al. 2016 and Bewick and Schmitz 2017). The phenotypic effects of cytosine methylation depend on the class and location of the methylation (Niederhuth et al. 2016; Bewick and Schmitz 2017). For example, transcriptional silencing of transposable elements can be achieved through the RNA-directed DNA methylation pathway (e.g. Matzke et al. 2015; Fultz et al. 2015) and is usually associated with an enrichment of mCHH as well as mCG and mCHG (Matzke et al. 2015; Bewick et al. 2016 and Bewick and Schmitz 2017; Hirsch and Springer 2017). Enrichment of mCHH as well as mCHG can also occur in genes and promoter regions, and is usually associated with gene silencing (Matzke et al. 2015; Neri et al. 2017). However, despite the fact that mCG is the most common form of cytosine methylation in the genomes of angiosperms (Niederhuth et al. 2016; Bewick and Schmitz 2017), the functional consequence of mCG enrichment in genes is not well understood.

Gene-body methylation (gbM) refers to the situation where mCG is enriched within the transcribed regions (coding and non-coding) of a gene, accompanied by a mCG depletion at the transcriptional start and termination sites and an overall mCHG and mCHH depletion within the gene (Bewick and Schmitz 2017). In plants, gbM is usually found in genes with intermediate expression level, which evolve slowly, and is often broadly conserved over millions of years of evolution (Zhang et al. 2006; Takuno and Gaut 2012, 2013; Bewick and Schmitz 2017). Genes with gbM tend to show stable expression both across tissues (Zhang et al. 2006; Zilberman et al. 2007; Takuno and Gaut 2012) and in different environmental conditions (Dubin et al. 2015), and a recent study in the outcrossing crucifer *Capsella grandiflora* identified the presence of gbM as a major predictor of *cis-*regulatory constraint (Steige et al. 2017). These results suggest that genes with gbM generally have a lower tolerance to variation in gene expression. Indeed, orthologues with conserved gbM between *A. thaliana* and *A. lyrata* exhibited significantly lower expression divergence than other genes, and changes in gbM levels tended to have occurred in lowly expressed (presumably functionally less important) genes (Takuno et al. 2017).

The long-term evolutionary conservation of gbM across orthologues further suggests that gbM might have a function; however, it is not currently clear what that function might be. The fact that some plant species lack gbM (e.g. Bewick et al. 2016) have led researchers to question whether gbM is essential (Zilberman 2017). Thousands of genes are the target of heterochromatinization in *A. thaliana*, where CG and CHG contexts are heavily methylated in their gene bodies, but the increase in BONSAI methylation 1 (IBM1) protein removes mCHG and histone H3 lysine 9 methylation (H3K9me) to keep such genes expressed, and as a consequence, mCG remains in expressed genes (Saze and Kakutani 2011). Thus, gbM could potentially be a by-product of this molecular process, which could itself be subject to evolutionary constraint (Takuno and Gaut 2013; Bewick and Schmitz 2017).

While experimental studies seem to exclude a role of gbM in gene silencing (e.g. Jones 2012; Bewick et al. 2016), it is possible that gbM is associated with changes in other aspects of transcriptional regulation. For instance, it has been hypothesised that gbM is associated with reduced transcriptional noise or erroneous transcription (Bird 1995; Suzuki et al. 2007; Zilberman et al. 2007, 2008; Huh et al. 2013; Neri et al. 2017). However, a previous study in plants using expression data from tissue samples found little evidence for an impact of gbM on erroneous transcription (Bewick et al. 2016), but a reanalysis of the same tissue samples revealed a small but significant effect of gbM on gene expression, indicating a potentially homeostatic role of gbM (Muyle and Gaut 2018). Despite some differences in the available enzymes and pathways for DNA methylation in plants and animals, gbM is very similar in plants and animals regarding the methylation patterns in gbM genes, and in both, gbM genes tend to have an intermediate expression level (Zemach and Zilberman 2010; Zilberman 2017). A recent study in humans suggested a possible role of gbM in reducing transcriptional noise (Huh et al. 2013). Additionally, in *Arabidopsis thaliana*, gbM was also found to play an important role in preventing the accumulation of the H2A.Z histone in gene bodies, and such an accumulation was reported to increase the responsiveness of a gene to the environment (Coleman-Derr and Zilberman 2012; To and Kim 2014). Hence, gbM could present a way to maintain a consistent expression of genes by preventing an increase in their responsiveness. However, so far, no study has comprehensively tested whether the presence of gbM is associated with altered expression noise and expression consistency in plants using single-cell data.

Although gbM could be linked to reduced gene expression noise, the amount of expression noise tolerated by a gene could also be affected by other genomic features. For instance, in yeast, dosage-sensitive and essential genes were found to tolerate less expression noise than other genes (Lehner 2008). Hence, to fully understand the relationship between gbM and gene expression noise, it is crucial to disentangle direct and indirect effects, which could result from a “hidden” third genomic feature affecting both gbM and gene expression noise simultaneously.

In this study, we investigate the potential role of gbM in reducing gene-specific transcriptional noise and intron retention as well as in maintaining consistent gene expression across cells. For this purpose and to avoid artefacts resulting from comparing cells in different cell stages, we analyse single-cell RNA-sequencing (RNAseq) data from root quiescent centre (QC) cells of *Arabidopsis thaliana* (Efroni et al. 2015), which are known to have relatively low mitotic activity (Nawy et al. 2005). We specifically ask if the observed relationships between gbM and gene expression noise, expression consistency as well as intron retention level are due to direct effects, or indirect effects of other correlated genomic factors.

## Materials and Methods

### Single-cell RNAseq data

We downloaded single-cell RNAseq data from 20 *A. thaliana* Col-0 root QC cells, generated by Efroni et al. (2015), from the National Center for Biotechnology Information (NCBI accession number GSE46226; SRA accession number: SRX730997–SRX731016). The RNA reads were trimmed with Trimmomatic 0.36 (Bolger et al. 2014) and mapped to the TAIR10 *A. thaliana* reference genome (https://www.arabidopsis.org/download/index-auto.jsp?dir=%2Fdownload_files%2FGenes%2FTAIR10_genome_release, December 2017) with STAR 2.5.3a (Dobin et al. 2013) following the recommended settings modifications necessary for downstream analyses with Cufflinks. Based on the mapped RNAseq reads, gene expression was quantified using Cufflinks 2.2.1 with default settings and treating each single-cell dataset as an individual sample with no replicates while running Cuffdiff (Trapnell et al. 2010, 2013; Roberts et al. 2011a, b). These analyses were done using the *A. thaliana* TAIR10 genome annotation GFF file downloaded from the Genome Portal of the Department of Energy, Joint Genome Institute (Nordberg et al. 2014).

### Identification of gbM

To distinguish between gbM and unmethylated genes, we analysed published whole-genome bisulfite-sequencing (bisulfite-seq) dataset from two biological replicates of root samples from *A. thaliana* Col-0 (Seymour et al. 2014; ENA accession number: PRJEB6701). The *A. thaliana* bisulfite-seq paired-end reads were trimmed with Trimmomatic 0.36 (Bolger et al. 2014) and mapped to the TAIR10 *A. thaliana* reference genome, using bismark 0.18.2 (Krueger and Andrews 2011). The methylation status of each gene was evaluated by comparing the CG, CHG and CHH DNA methylation level of the gene-body (from the transcription start to termination site, including both exons and introns) to the average DNA methylation level of all genes, based on a binomial probability distribution as in Takuno and Gaut (2012) and Takuno et al. (2017). Briefly, because a CHG and CHH methylation depletion is typical for gbM genes (Bewick and Schmitz 2017), genes which had a significantly higher CHG and/or CHH DNA methylation level than the genomic average (*P* value <0.05) were labelled highly methylated non-gbM genes (Takuno and Gaut 2012 and Takuno et al. 2017) and excluded from further analyses. The remaining genes were split in the following three groups: unmethylated genes, gbM genes and ambiguous genes if their CG DNA methylation level was significantly lower (*P* value >0.95), significantly higher (*P* value <0.05) or similar to the genomic average, respectively, following Takuno and Gaut (2012) and Takuno et al. (2017). Only genes that were identified as gbM or unmethylated in both replicate bisulfite-seq samples were considered as gbM or unmethylated in this study, respectively. Overall, out of 21,754 genes with unambiguous methylation status in both samples, 21,616 showed concordant methylation status. Additionally, we evaluated if the use of DNA methylation in the gene body, including introns, had a major impact on the methylation status assessment by reclassifying all genes into gbM and unmethylated genes using the method described above, but this time only using DNA methylation in exons. A total of 94% of gbM and 96% of unmethylated genes were classified identically in the second approach. Furthermore, only 0.1% of gbM and <0.1% of unmethylated genes had a strictly conflicting classification, meaning that these genes were classified as gbM, unmethylated or highly methylated non-gbM genes using the second approach, but their classification differed between our two approaches. The remaining genes were identified as intermediately methylated using the second approach. We also examined our expectation that gbM is highly correlated across different datasets and tissues in *A. thaliana* by analysing an additional whole-genome bisulfite-sequencing dataset from an *A. thaliana* Col-0 leaf sample generated by an independent study (Bewick et al. 2016; GEO accession number: GSE75071). After classifying all genes following the approach described above, 90% of gbM and 96% of unmethylated genes were classified identically in the second dataset, with only 0.6% of gbM and 0.3% of unmethylated genes having a strictly conflicting classification in the two datasets, meaning that they were assessed as gbM, unmethylated or highly methylated non-gbM genes in the second dataset, but their classification differed in the two datasets. The rest of the genes were identified as intermediately methylated in the second dataset.

### Gene expression noise quantification

Gene expression noise was estimated from the fragments per kilobase of transcripts per million mapped fragments (FPKM) tracking output from Cufflinks. We used the measure of stochastic gene expression (F*) defined by Barroso et al. (2018) as a measure for gene expression noise. This measure is intended to correct for the fact that expression level and variance are expected to be correlated (Barroso et al. 2018). Briefly, stochastic gene expression is defined as the ratio between the observed variance (*σ*^2^) and the expected variance given the mean (*μ*) expression level of a given gene over all single cells (Barroso et al. 2018). The expected variance is given by the lowest degree polynomial regression modelling log (*σ*^2^) as a function of log (*μ*), and as a result, F* is not correlated with the mean expression level (Barroso et al. 2018). In our case, the observed correlation between expression level and variance (Kendall’s rank correlation test *τ* = 0.887, *P* value <2.2 × 10^−16^) was removed when using a polynomial regression of the third degree to estimate log (*σ*^2^). The correlation between F* and *μ* was tested using a Kendall’s rank correlation test (Barroso et al. 2018), which revealed no significant correlation between F* and *μ* (Kendall’s rank correlation test *τ* = 0.0113, NS) confirming the independence of F* and mean expression level. Only genes that had an expression level with a log (FPKM + 1) > 1.5 in at least one sample were used in this study, as suggested by Barroso et al. (2018). In a second attempt to quantify gene expression noise without correcting for the effects of the expression level, we defined transcriptional noise (*F*ʹ) as the coefficient of variation (standard deviation/mean) of the gene FPKM tracking following Yin et al. (2009) and Huh et al. (2013). This was done to control for potential artefacts introduced by the expression level correction of the F* expression noise measure.

### Gene expression consistency

To investigate if gbM genes were more consistently expressed in the root QC cells than unmethylated genes, we defined the expression consistency of the genes included in this study (see above) as the number of replicate single cells in which the gene was detected as expressed (FPKM > 0). Hence, the expression consistency of a gene is high when that gene is expressed in many samples indicating a consistent expression of the gene in the root QC cells. Gene expression consistency as used here provides similar information to the measure of gene expression noise, but these two metrics are not completely identical. The main difference is that expression consistency only considers if a gene is expressed or not, meaning that if two genes are expressed in exactly the same number of samples they are going to have the same expression consistency regardless of differences in expression levels between samples. However, gene expression noise discriminates between genes that are expressed in the same number of samples, but with varying expression level differences among samples. Importantly, as defined here, the measure of expression consistency is expected to be biased toward generating higher estimates for highly expressed and long genes. To account for this effect, we included mean expression level and gene length as explanatory variables in our statistical analyses of expression consistency.

### Statistical analyses of gene expression noise and consistency

Differences in the expression noise level and other genomic features between gbM and unmethylated genes were first examined with a two-sided Mann–Whitney *U* test and the relationship between the other genomic features and expression noise were examined with Spearman’s rank-order correlation tests and Mann–Whitney *U* tests. We used an analysis of covariance (ANCOVA) to assess the effect of gbM after accounting for the effects of other genomic features. Similar ANCOVA analyses were done with both expression noise and expression consistency as response variables. In these analyses, we log transformed a variable if the log transformation led to an increase in *R*^2^, except for the mean expression level when predicting F* in order to avoid introducing correlations between F* and the mean expression level through transformations. We centred and scaled our variables before ANCOVA analyses and performed a Bayesian information criterion (BIC) model averaging model selection to find the best model. The effect of multicollinearity of the variables was investigated using the variance inflation factor (vif) and √vif < 2 was required for each variable. These analyses were run in R 3.4.3 (R Core Team 2017) using the “car” (Fox and Weisberg 2011) and “MuMIn” (Bartoń 2018) packages. To further confirm that our results are robust to multicollinearity, we ran a linear regression analyses using gbM and the principal components of the other genomic features of interest identified by our ANCOVA as predictor variables, following Drummond et al. (2006). As the principal components are by definition orthogonal, this reduces problems with multicollinearity. For these analyses, we used the “pls” R package (Mevik et al. 2016).

In our analyses of expression noise and expression consistency, we included nine additional genomic features, which could affect the observed expression noise of a gene. These features are: gene length, mean expression level, predicted gene lethality, the ratio of non-synonymous and synonymous divergence (*Ka*/*Ks*) between *A. thaliana* and *A. lyrata*, co-expression module size, gene duplicates retained in the *A. thaliana* genome from the α and βγ whole-genome duplication (WGD), expression breadth and presence/absence of tandem duplicates, as estimated by Lloyd et al. (2015). Gene expression noise was previously described to be negatively correlated with gene length, lethality and mean expression level (Lehner 2008; Huh et al. 2013; Barroso et al. 2018). Additionally, selection preventing noise propagation within gene networks was identified to reduce the observed amount of expression noise in mouse (Barroso et al. 2018); hence, the co-expression module size of a gene, as a proxy for network centrality, could also impact the amount of expression noise tolerated by a gene. Furthermore, genes experiencing strong selection could also be less tolerant to high expression noise levels. Here, we regard *Ka*/*Ks* value between *A. thaliana* and *A. lyrata* as a proxy for selective constraint, in order to be able to account for this effect. Similarly, genes that are retained in duplicate for prolonged periods of time (like duplicates retained from the α and βγ WGD) or that can tolerate tandem duplication, likely experience different selective pressures from single-copy genes (Maere et al. 2005; Li et al. 2016), and could also exhibit different expression noise distributions than other genes. Finally, genes with different expression breadth show different rates of protein evolution (Slotte et al. 2011) and thus expression breadth could also affect variation in the observed amount of expression noise of a gene. Consequently, we included published information on gene lethality, *Ka*/*Ks*, co-expression module size, gene duplicates retained in the *A. thaliana* genome from the α and βγ WGD, expression breadth and presence/absence of tandem duplications in *A. thaliana* (Lloyd et al. 2015) in our analyses. Finally, we compared the evolutionary rate of gbM and unmethylated genes based on estimates of non-synonymous divergence (*Ka*) between *A. thaliana* and *A. lyrata* obtained by Takuno et al. (2017).

### Intron retention analyses

To quantify the number of reads mapping to introns, we modified the *A. thaliana* genome annotation to mark all positions of each non-overlapping gene, which were not part of any exon or UTR, as intron. Then, all gene annotation entries were removed except “gene”, “mRNA” and “intron”, and all introns were relabelled as “exon” as suggested by Bewick et al. (2016). This modified genome annotation was then used to map the single-cell RNAseq reads to the reference genome using STAR 2.5.3a (Dobin et al. 2013). Then, the Bioconductor package Rsubread 1.28.1 (Liao et al. 2013) was used to count the number of reads mapping to introns as well as to count the number of reads that mapped to the respective exons of the gene when we mapped the RNAseq dataset using the regular annotation file. These counts were then added to generate a total number of reads mapping to each gene. To test whether gbM had an effect on the number of reads mapping to introns, we first calculated the intron FPKM and total FPKM of each gene based on our count data and only included genes in our model which fulfilled log (total FPKM + 1) > 1.5, identically to our gene filtering for the gene expression noise analyses. We then performed an ANCOVA as described above with the intron FPKM as the response variable. We included two additional variables, total intron length and intron number, which are expected to influence the observed intron FPKM of a gene.

## Results

### GbM genes show more consistent expression and less expression noise than unmethylated genes

We detected a total of 11,659 genes that had an expression level sufficiently high in at least one of the single-cell RNAseq samples to be included in this study (see Materials and methods). Of these, 3799 and 5176 genes were identified as gbM and unmethylated genes, respectively, whereas the rest were either highly methylated non-gbM or ambiguous (see Materials and methods). A total of 13% of genes were expressed in all 20 single-cell replicates, whereas only 4.6% of the genes were expressed in only one replicate.

**Fig. 1:**
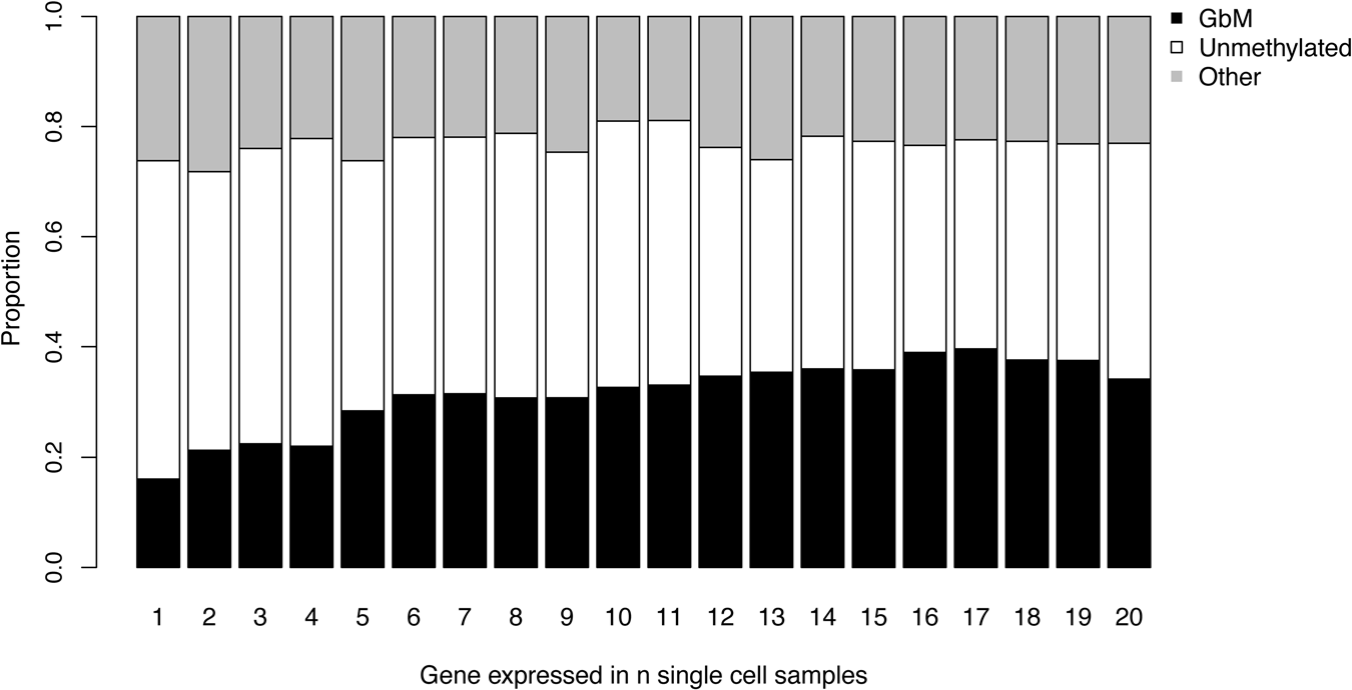
Proportion of gbM (black), unmethylated (white) and other (highly methylated non-gbM and ambiguous; grey) genes found to be expressed in *n* number of single-cell samples of *A. thaliana* QC cells

Comparing gbM to unmethylated genes revealed that gbM genes had significantly lower expression noise than unmethylated genes, using both noise measures (F* and *F*ʹ, Table 1). Considering gene expression consistency (the number of single cells in which a gene was expressed), gbM genes had a significantly higher gene expression consistency than unmethylated genes. Sixteen and 58% of the genes found to be expressed in only one single-cell sample were gbM and unmethylated, respectively, whereas 34 and 43% of the genes found to be expressed in all single-cell sample were gbM and unmethylated, respectively (Table 1 and Fig. 1). Additionally, we found gbM genes to be significantly longer than unmethylated genes (Table 1) and to have intermediate expression levels (supplementary Fig. S1). Finally, significantly fewer gbM genes were retained from the α and βγ WGD or tandem duplicated and gbM genes had on average a significantly higher co-expression module size and expression breadth (number of tissues in which a gene was found expressed; Lloyd et al. 2015), than unmethylated genes (Table 1). The difference in the gene expression breadth between gbM and unmethylated genes was due to a higher proportion of the gbM genes (82.4 vs. 72% unmethylated genes) having a maximal expression breadth.

**Table 1:** Observed differences between gbM and unmethylated genes regarding various genomic features based on a two-sided Mann–Whitney *U* test

Genomic features	gbM	Unmethylated	*P* value
Estimated stochastic gene expression F*	0.96	1.11	<2 × 10^−16^
Estimated transcriptional noise *F*ʹ	2.94	3.10	3.16 × 10^−14^
Expression consistency	14	11	<2 × 10^−16^
Mean expression level	8.64	13.85	<2 × 10^−16^
Gene length (bp)	3450	1783	<2 × 10^−16^
Lethal gene	23.4%	13.1%	<2 × 10^−16^
*A. lyrata* homologue *Ka*/*Ks*	0.156	0.153	0.179
*A. thaliana* vs. *A. lyrata Ka* estimate	0.017	0.019	3.15 × 10^−5^
Co-expression module size	19	14	<2 × 10^−16^
Expression breadth	64	64	<2 × 10^−16^
Gene duplicates retained from the α WGD	26.77%	33.06%	2.29 × 10^−10^
Gene duplicates retained from the βγ WGD	10.29%	13.56%	3.72 × 10^−6^
Tandem duplicated genes	7.6%	10%	1.05 × 10^−4^

The differences are shown as proportions for binary variables and as median values for non-binary variables. *P* values were corrected for multiple testing using a Benjamini and Hochberg *P* value adjustment

However, some of the features for which gbM and unmethylated genes differ were significantly correlated with each other (supplementary table S1), as is common for genomic data. Hence, gbM genes seem to be generally less noisy with respect to their expression and more consistently expressed than unmethylated genes (Table 1 and Fig. 1). But this could be due to confounding factors that are correlated with both gbM and expression noise or consistency. Furthermore, both gene expression noise measures (F* and *F*ʹ) were negatively correlated with expression consistency (Spearman’s rank-order correlation test, *ρ* = −0.36 and *ρ* = −0.59, *P* value <2.2 ×10^−16^ and *P* value < 2.2 × 10^−16^, respectively).

### GbM is indirectly linked to expression noise and directly linked to expression consistency

To resolve the relationship between gbM and gene expression noise as well as expression consistency, we performed an ANCOVA on a set of 5637 genes, for which information on all the genetic features investigated in this study were available. If gbM has a direct effect on gene expression noise and/or expression consistency, then the observed significant correlation between gbM and gene expression noise as well as expression consistency should still be detectable when adjusting for effects of other genomic features.

The averaged ANCOVA model revealed no significant effect of gbM on either F* nor *F*ʹ, a low relative predictor importance of gbM and a BIC best model selection did not include gbM as predictor (supplementary table S2 and S3). Indeed, the best model (selected by BIC model selection) included only gene length, expression breadth and gene duplicates retained from the α WGD as well as expression level, gene length, expression breadth and gene duplicates retained from the α WGD as explanatory variables for F* and *F*ʹ, respectively (supplementary table S2 and S3). To investigate if the lack of a relationship between gbM and expression noise was due to a loss of power resulting from the downsampling of our data from 8975 genes for which gbM information was available to 5637 genes for which information on all the genomic features were available, we reran an ANCOVA and a BIC model selection, but this time only including genomic features for which information was available for all 8975 genes. This second averaged ANCOVA model also revealed no significant effect of gbM on gene expression noise (supplementary table S4); hence, the lack of a significant effect of gbM on expression noise in the first averaged ANCOVA model, including all genomic features studied, was likely not due to a loss of detection power. Rather, gene length, expression breadth and gene duplicates retained from the α WGD seem to indirectly link gbM to gene expression noise through the strong correlation of gbM with these genomic features. Similar results were obtained for both the stochastic gene expression F* and the transcriptional noise *F*ʹ; hence, this result is not due to the way in which gene expression noise was estimated. However, the variance explained by our models was generally low (*R*^2^ = 0.0261 and *R*^2^ = 0.1016 for F* and *F*ʹ, respectively). To further confirm our findings, we reanalysed the dataset using principal components of our genomic features to avoid multicollinearity. This confirmed that, when considering the genomic features identified as significantly correlated with F* as well as *F*ʹ by the ANCOVA, gbM was not significantly correlated with gene expression noise (supplementary table S5 and supplementary Fig. S2 and S3). Finally, to investigate potential effects of using a different dataset or tissue sample for classifying genes as gbM, we reran our analyses with only the genes assessed as gbM and unmethylated using both the Seymour et al. (2014) and Bewick et al. (2016) bisulfite-seq datasets. This analysis, with a stricter gene set of 2236 gbM and 2828 unmethylated genes, revealed similar results to the previous analyses, showing no significant effect of gbM on gene expression noise (supplementary table S6).

The ANCOVA analysis of the relationship between gbM and gene expression consistency revealed a significant positive effect of gbM on gene expression consistency even when adjusting for other genomic features and gbM was kept in the best model (based on a BIC model averaging model selection), which could explain a substantial fraction of the observed variation (*R*^2^ = 0.55; Table 2 and supplementary table S7). Hence, gbM is associated with gene expression consistency even after adjusting for the effects of other genomic features. In addition to gbM, gene length, expression level, expression breadth and gene duplicates retained from the α WGD were kept in our best model and all had a significantly positive effect on expression consistency (Table 2 and supplementary table S7). Highly similar results were obtained in a linear regression analyses using gbM and principal components of the other genomic features, previously identified as significantly correlated with expression consistency, as predictor variables (supplementary table S8 and supplementary Fig. S4). Finally, we reanalysed the relationship between gbM and gene expression consistency by only using genes assessed as gbM and unmethylated using two independent bisulfite-seq datasets (Seymour et al. 2014; Bewick et al. 2016). These analyses also indicated an effect of gbM on gene expression consistency after adjusting for the effects of other genomic features (supplementary table S9).

**Table 2:** Best ANCOVA model of gene expression consistency based on a BIC model averaging model selection (*R*^2^ = 0.55; *n* = 5 637)

Genetic features	Gene expression consistency			
	Coefficients	Sum of squares	*F* value	*P* value
Log (mean expression level)	4.222	97,160	6 325.4	<2 × 10^−16^
Log (gene length)	1.373	6690	435.6	<2 × 10^−16^
gbM	0.457	180	11.7	6.34 ×10^−4^
Expression breadth	0.531	1538	100.1	<2 ×10^−16^
Gene duplicates retained from the α WGD	0.513	312	20.4	6.61 ×10^−6^

### Fewer RNAseq reads map to gbM gene introns than to unmethylated gene introns

Previously, Bewick et al. (2016) found no evidence for an impact of gbM on the level of introns retained in the spliced RNA molecule, when comparing gbM and unmethylated genes using tissue RNA samples. To test if the use of a single-cell RNA dataset could uncover subtle differences in the intron retention levels of gbM and unmethylated genes, we estimated the amount of reads mapping to introns similarly to Bewick et al. (2016; see Materials and methods) and ran ANCOVA analyses to elucidate which genomic features could best predict the amount of reads mapping to introns.

Here, we detected a total of 8133 non-overlapping genes that had an expression level sufficiently high in at least one of the single-cell RNAseq samples to be included in the intron retention analysis (see Materials and methods). Of these, 3477 and 4656 genes were classified as gbM and unmethylated genes, respectively, whereas the rest were either highly methylated non-gbM or ambiguous (see Materials and methods). The model averaging ANCOVA model selection revealed a significant negative effect of gbM on the number of reads mapping to the introns of a gene (Table 3 and supplementary table S10). Additionally, the expression level, total intron length and the number of introns had a significant positive effect, whereas the gene length and expression breadth had a significant negative correlation with the number of reads mapping to introns (Table 3 and supplementary table S10). Similar results were obtained when using principal components of the genomic features as well as gbM as predictor variables (supplementary table S11 and supplementary Fig. S5). The results further remained unchanged when classifying genes as gbM or unmethylated using two independent bisulfite-seq datasets (Seymour et al. 2014; Bewick et al. 2016) (supplementary table S12).

**Table 3:** Best ANCOVA model of log (intron FPKM) based on a BIC model averaging model selection (*R*^2^ = 0.731; *n* = 4376)

Genetic features	Log (intron FPKM)			
	Coefficients	Sum of squares	*F* value	*P* value
Log (gene FPKM)	1.56	9 974.6	9 751.4	<2 × 10^−16^
Log (gene length)	−0.83	909.7	889.3	<2 × 10^−16^
Log (total intron length)	0.46	256.1	250.4	<2 ×10^−16^
Log (intron number)	0.89	973.8	952	<2 ×10^−16^
gbM	−0.13	11.8	11.6	6.81 ×10^−4^
Expression breadth	−0.08	29.5	28.9	8.17 × 10^−8^

## Discussion

### GbM is not associated with reduced expression noise in *A. thaliana* root QC cells

We first found that gbM genes had lower expression noise than unmethylated genes, in line with the proposed role of gbM in reducing expression noise (Bird 1995; Suzuki et al. 2007; Huh et al. 2013) and with previous findings in human brain and blood tissue (Huh et al. 2013). However, gbM and unmethylated genes also differed with respect to a number of genomic features. For instance, as previously reported (Takuno and Gaut 2012, 2013; Bewick and Schmitz 2017), gbM genes were significantly longer than unmethylated genes and had an intermediate expression level (Table 1 and supplementary Fig. S1). Additionally, we found gbM genes to have on average a significantly higher expression breadth and a significantly higher proportion of gbM genes were lethal (Table 1), which is in concordance with literature (Zhang et al. 2006; Takuno and Gaut 2012). GbM genes also differed significantly from unmethylated genes regarding the presence of tandem duplications and retained copies from the α and βγ WGD (Table 1). Surprisingly, we did not observe a significant difference in the *Ka*/*Ks* of gbM and unmethylated genes despite previous reports of gbM genes being evolutionarily more constrained than unmethylated genes (e.g. Takuno and Gaut 2012). However, Takuno and Gaut (2012) were more stringent regarding the estimation of *Ka*/*Ks* by requesting the aligned sequences to include at least 100 synonymous sites, which was not requested for the *Ka*/*Ks* estimation in the dataset used in this study (Lloyd et al. 2015). Hence, a less precise estimation of *Ka*/*Ks* could have affected our power to detect this correlation in our analyses. Furthermore, although gbM genes do not seem to be more constrained, they do evolve at a lower rate in terms of *Ka* (Table 1), in line with the observations in Takuno and Gaut (2012).

Correcting for the effects of other genomic features revealed that gbM genes did not differ from unmethylated genes with respect to their expression noise. This result was robust to different expression noise estimation. Additionally, these results were robust to the effect of downsampling. However, the variance explained by our models was generally low (*R*^2^ = 0.0261 and *R*^2^ = 0.1016 for F* and *F*ʹ, respectively), indicating that other features not included in this study could be important to consider when analysing variation in expression noise in *A. thaliana* root QC cells. Indeed, microRNAs are known to accelerate the mRNA degradation of targeted genes and were hypothesised to play a role in increasing gene expression precision trough noise reduction (e.g. Bartel and Chen 2004; Schmiedel et al. 2015) and, in yeast, dosage sensitivity was also reported to affect gene expression noise (Lehner 2008).

Nevertheless, our gene expression noise analyses revealed that genes with a high expression breadth and genes retained from the α WGD were less noisy than other genes (supplementary table S4). The observation that genes with duplicates retained from the younger α WGD (Bowers et al. 2003) were less noisy than other genes, but genes with duplicates retained from the older βγ WGD (Bowers et al. 2003) did not differ from other genes with respect to their expression noise, and is in line with the gene balance hypothesis, which predicts that gene dosage balance has an important impact on fitness (Birchler and Veitia 2012). Indeed, in plants, unlike other gene duplicates originating from a WGD, duplicates that are dosage sensitive tend to be retained during intermediate evolutionary periods before being ultimately lost (Li et al. 2016). Hence, in *A. thaliana*, genes retained from the α WGD could harbour disproportionately more dosage-sensitive genes than genes retained from the βγ WGD or tandem duplicated genes. This could explain why genes with duplicates retained from the α WGD were less noisy than other genes, but the observed expression noise of other duplicated genes was not reduced. Identically, genes with a high expression breadth could primarily be key genes, like housekeeping genes, and could be under selection to minimise their expression noise.

### GbM genes are more consistently expressed than unmethylated genes

Analysing the relationship between gbM and gene expression consistency revealed that genes with gbM were significantly more consistently expressed than unmethylated genes even after adjusting for effects of other genomic features (Table 2). This result is in line with previous results describing a negative correlation between gbM and gene responsiveness in *A. thaliana* (Aceituno et al. 2008) and with observations of Coleman-Derr and Zilberman (2012), who postulated that gbM could have a function in preventing the accumulation on the H2A.Z histone in the body of a gene, which is associated with an increase in the responsiveness of a gene (Coleman-Derr and Zilberman 2012; To and Kim 2014). Additionally, the loss of H2A.Z histones from the gene body causes a change in the expression regulation of a gene indicating different regulatory requirements for responsive and housekeeping genes (Coleman-Derr and Zilberman 2012). However, Bewick et al. (2016) investigated a potential function of gbM in preventing H2A.Z histone from accumulation in gene bodies using *met1* mutant *A. thaliana* plants, in which gbM was missing, and found no differences in the distribution patterns of the H2A.Z histone in genes which lost gbM. Hence, this could indicate that gbM is not causing a histone H2A.Z depletion and an increased expression consistency, but that gbM is a by-product of a histone H2A.Z depletion (Bewick et al. 2016). Nevertheless, our results are compatible with these observations since we found a correlation between gbM and gene expression consistency, which does not exclude the possibility that gbM is induced by an increased gene expression consistency through the depletion of H2A.Z histones.

Additionally, our analyses also revealed that genes with high expression breadth and gene with duplicates retained from the α WGD were significantly more consistently expressed than other genes (Table 2), as might be expected for dosage-sensitive genes and housekeeping genes.

### GbM potentially enables a more accurate RNA splicing trough reduced intron retention

Unlike Bewick et al. (2016), here, we find a correlation between gbM and the amount of reads mapping to the introns of a gene, which points to a role of gbM in reducing RNA mis-splicing in the form of intron retention. In their study, Bewick et al. (2016) compared wild-type *A. thaliana* Col-0 individuals to *met1* mutant *A. thaliana* plants, in which gbM was missing, and found no evidence for an impact of gbM on intron retention. Eliminating gbM is indeed a good approach to study such a hypothesis; however, it is possible that some redundancies in the function of different epigenetic modifications could compensate for the absence of gbM in the mutant plants (Bewick et al. 2016). Indeed, H3K36me3 chromatin modifications, for example, were previously shown to impact splicing regulation (Lin and Workman 2011), and Bewick et al. (2016) tried to account for the effect of H3K36me3 chromatin modifications on mis-splicing by studying mutants that lacked gbM and H3K36me3, but found no evidence for differences in mRNA splicing between mutants and wild-type (Bewick et al. 2016). Nevertheless, it is still possible that other epigenetic modifications could compensate for the lack of gbM in the mutant plants, which resulted in wild-type-like RNA splicing. However, it is also possible that the observed differences in the amount of reads mapping to the introns of gbM and unmethylated genes (Table 3) could be the result of some other processes and not because of lower intron retention levels. Indeed, our analysis revealed that the amount of reads mapping to a gene, the total intron and gene length as well as the number of introns were significantly correlated with the number of reads mapping to the introns of a gene, which was expected since RNA samples will inevitably contain un-spliced RNA (La Manno et al. 2018). Hence, the amount of reads originating from introns of un-spliced reads is expected to depend on these characteristics of the gene. But the amount of un-spliced RNA in a sample is also expected to be affected by transcriptional dynamics (La Manno et al. 2018), and the proportion of un-spliced RNA observed could differ between genes, for which transcription started shortly before sampling and for those which were continuously expressed before sampling (La Manno et al. 2018). Hence, the observation that gbM genes have fewer reads mapping to their introns than unmethylated genes (Table 3), can either be explained by a lower level of un-spliced RNA derived from gbM genes in the QC cells or by a lower level of introns retained in the spliced RNAs of gbM genes. Disentangling these two explanations is very difficult based only on RNAseq data. Nevertheless, a lower level of introns retained in the spliced RNAs of gbM genes would be in line with previous suggestions that gbM could enable more accurate transcription (Regulski et al. 2013; Neri et al. 2017). Additionally, genes with a high expression breadth had fewer reads mapping to their introns than genes with low expression breadth (Table 3), indicating that selection could act on reducing intron retention in potentially key genes.

### GbM as result of gene regulation

Lastly, there is also the possibility that gbM itself is driven by some specificities of gene regulation. For example, Secco et al. (2015) reported methylation modifications after stress-induced gene expression changes due to phosphate deprivation in rice, but most of these methylation modifications occurred in transposable elements close to upregulated genes and similar experiments showed only a restricted stress induced methylation modification in *Arabidopsis* (Secco et al. 2015). Nevertheless, it could be possible that gbM is induced through regulatory processes, for instance, affecting gene expression consistency, which we found to be correlated with gbM. To investigate this possibility, we used a linear regression model with a BIC model selection to evaluate which genomic features should be included in a model predicting gbM. We used all genomic features as predictor in a single regression analyses except *F*ʹ and the intron number of a gene, which violated our requirement of √vif < 2. This analysis revealed that the best model to predict gbM includes gene length, mean expression level, total intron length, co-expression module size and expression breadth (supplementary table S13). This indicates that when it comes to predicting gbM, neither gene expression noise nor gene expression consistency, nor indeed the number of reads mapping to the introns of a gene, are essential genomic features to consider, at least in our analyses of this dataset. Nevertheless, it is possible that a histone H2A.Z depletion drives gbM (Bewick et al. 2016) and an increased gene expression consistency (Coleman-Derr and Zilberman 2012). Hence, gbM and gene expression consistency could be indirectly linked together through the distribution patterns of the H2A.Z histone.

## Conclusion

Despite being intensively studied in the recent years, it is still unclear if gbM has a function. Several hypotheses have been postulated regarding potential functions of gbM and, here, we used single-cell RNAseq data from root QC cells of *A. thaliana* to test these hypotheses. We investigated a possible function of gbM in reducing expression noise (Bird 1995; Suzuki et al. 2007; Huh et al. 2013), but found no support for this hypothesis. However, our analyses revealed that gbM genes were expressed on average in more single-cell replicates than unmethylated genes, even after correcting for the effect of expression level and gene length, indicating an involvement of gbM in the maintenance of a consistent gene expression either as antagonist of H2A.Z histones (Coleman-Derr and Zilberman 2012; To and Kim 2014) or as a by-product of H2A.Z histone depletion (Bewick et al. 2016). Finally, we investigated the hypothesis that gbM enables more accurate RNA splicing (Regulski et al. 2013; Neri et al. 2017) and found lower levels of RNAseq reads originating from introns from gbM genes, which could be a hint toward a lower intron retention level in the mRNAs of gbM genes or toward a lower proportion of un-spliced gbM gene RNAs. Here, we could not disentangle these two explanations, but further studies on intron retention levels could give new insights on the function of gbM. Our results are important for an improved understanding of the interplay between methylation and gene expression variation across plant genomes.

## Supplementary information

Supplementary Material
