# Supplementary material for: Single-cell expression noise and gene-body methylation in Arabidopsis thaliana
Source: Heredity (Edinb). 2019 Jan 16;123(2):81–91. doi: 10.1038/s41437-018-0181-z (PMC6781109; doi:10.1038/s41437-018-0181-z)
Supplement: Supplementary file 1 — Supplementary Material [file 41437_2018_181_MOESM1_ESM.pdf]

Supplementary Material

Single cell expression noise and gene-body methylation in *Arabidopsis thaliana*

Robert Horvath[1], Benjamin Laenen[1], Shohei Takuno[2], Tanja Slotte[1]

[1]Department of Ecology, Environment and Plant Sciences, Science for Life laboratory, Stockholm University, SE-106 91 Stockholm, Sweden

[2]Department of Evolutionary Studies of Biosystems, SOKENDAI (The Graduate University for Advanced Studies), Hayama, Kanagawa, Japan

*Corresponding authors:

Robert Horvath: robert.horvath@su.se

Tanja Slotte: Tanja.Slotte@su.se

Supplementary tables

Table S1. Observed correlations of F*, F’ as well as the expression consistency and various genomic features based on a two-sided Spearman’ rank-order correlation test and a two-sided Mann-Whitney U-test for binary variables. *P*-value were corrected for multiple testing using a Benjamini and Hochberg *p*-value adjustment.

**Genetic features**	**Estimated stochastic gene expression F***		**Estimated transcriptional noise F´**		**Gene expression consistency**	
	***rho* or median difference**	***p*-value**	***rho* or median difference**	***p*-value**	***rho* or median difference**	***p*-value**
Mean expression level	0.02126263	0.066	-0.3078724	$< 2*10^{-16}$	0.7157376	$< 2*10^{-16}$
Gene length	-0.1401393	$< 2*10^{-16}$	-0.1167594	$< 2*10^{-16}$	0.1825358	$< 2*10^{-16}$
Lethal gene	-0.044	0.24	-0.029	0.082	1	0.23
A. lyrata homolog *Ka/Ks*	0.06814572	$1.54*10^{-7}$	0.1244503	$< 2*10^{-16}$	-0.1591528	$< 2*10^{-16}$
Co-expression module size	-0.05019564	$1.57*10^{-5}$	-0.04837808	$2.74*10^{-5}$	0.02263742	0.058
Expression breadth	-0.1380663	$< 2*10^{-16}$	-0.2085376	$< 2*10^{-16}$	0.244334	$< 2*10^{-16}$
Gene duplicates retained from the α WGD	-0.081	$2.89*10^{-6}$	-0.121	$8.69*10^{-12}$	2	$7.2*10^{-15}$
Gene duplicates retained from the $\beta\gamma$ WGD	-0.007	0.37	-0.018	0.097	2	0.0013
Tandem duplicated genes	0.022	0.72	0.006	0.24	0	0.85

Table S2. Model averaged ANCOVA of F* as well as F' with all genomic features studied (R^2=0.0199 and R^2=0.0925, respectively; n=5 637). Genomic features which were kept in the best model generated by a BIC model averaging model selection are indicated in the last column.

**Genetic features**	**Estimated stochastic gene expression F***						**Genetic features**	**Estimated transcriptional noise F´**					
	Coefficients	Adjusted Standard error	Z value	Pr(>|z|)	Relative variable importance	Included in best model		Coefficients	Adjusted Standard error	Z value	Pr(>|z|)	Relative variable importance	Included in best model
Mean expression level	-2.344e-04	2.063e-03	0.114	0.910	0.02	No	Log (mean expression level)	-0.2533399	0.0125023	20.26	$< 2*10^{-16}$	1.00	Yes
Log (gene length)	-8.315e-02	9.268e-03	8.972	$< 2*10^{-16}$	1.00	Yes	Log (gene length)	-0.1189630	0.0128415	9.264	$< 2*10^{-16}$	1.00	Yes
gbM	-9.619e-05	2.864e-03	0.034	0.973	0.01	No	gbM	-0.0004216	0.0052589	0.080	0.936	0.02	No
Lethal gene	-4.820e-05	2.885e-03	0.017	0.987	0.01	No	Lethal gene	-0.0002072	0.0044945	0.046	0.963	0.01	No
A. lyrata homolog *Ka/Ks*	1.200e-03	5.041e-03	0.238	0.812	0.07	No	*A. lyrata* homolog *Ka/Ks*	0.0101158	0.0168964	0.599	0.549	0.30	No
Co-expression module size	-4.920e-04	3.122e-03	0.158	0.875	0.04	No	Co-expression module size	-0.0100867	0.0169814	0.594	0.553	0.30	No
Expression breadth	-6.338e-02	9.247e-03	6.854	$< 2*10^{-16}$	1.00	Yes	Expression breadth	-0.1165587	0.0127784	9.122	$< 2*10^{-16}$	1.00	Yes
Gene duplicates retained from the α WGD	-9.190e-02	2.030e-02	4.527	$< 2*10^{-16}$	1.00	Yes	Gene duplicates retained from the α WGD	-0.1242538	0.0270938	4.586	$4.5*10^{-6}$	1.00	Yes
Gene duplicates retained from the $\beta\gamma$ WGD	7.494e-05	3.284e-03	0.023	0.982	0.01	No	Gene duplicates retained from the $\beta\gamma$ WGD	0.0001605	0.0045869	0.035	0.972	0.01	No
Tandem duplicated genes	-1.728e-05	4.079e-03	0.004	0.997	0.01	No	Tandem duplicated genes	0.0001026	0.0055777	0.018	0.985	0.01	No

Table S3. Best ANCOVA model of F* as well as F' based on a BIC model averaging model selection (R^2=0.0261 and R^2=0.1016, respectively; n=5 637).

**Genetic features**	**Estimated stochastic gene expression F***				**Genetic features**	**Estimated transcriptional noise F´**			
	Coefficients	Sum of squares	F value	Pr(>F)		Coefficients	Sum of squares	F value	Pr(>F)
Log (gene length)	-0.08327113	38.69	81.875	$< 2*10^{-16}$	Log (gene length)	-0.2537922	352.6	423.933	$< 2*10^{-16}$
Expression breadth	-0.06364979	22.66	47.961	$4.8*10^{-12}$	Log (mean expression level)	-0.1210657	80.4	96.698	$< 2*10^{-16}$
Gene duplicates retained from the α WGD	-0.09210845	10.12	21.415	$3.8*10^{-6}$	Expression breadth	-0.1192002	78.6	94.526	$< 2*10^{-16}$
					Gene duplicates retained from the α WGD	-0.1234328	18.1	21.779	$3.1*10^{-6}$

Table S4. Model averaged ANCOVA of F* as well as F' only including genomic features for which information was available for all genes (R^2=0.0153 and R^2=0.0972, respectively; n=8 975). Genomic features which were kept in the best model generated by a BIC model averaging model selection are indicated in the last column.

Genetic features	Estimated stochastic gene expression F*					
	Coefficients	Adjusted Standard error	Z value	Pr(>|z|)	Relative variable importance	Included in best model
Mean expression level	-1.492e-04	1.464e-03	0.102	0.919	0.02	No
Log (gene length)	-1.005e-01	7.319e-03	13.74	$< 2*10^{-16}$	1.00	Yes
gbM	-3.890e-04	3.774e-03	0.103	0.918	0.02	No
Lethal gene	-2.473e-04	3.138e-03	0.079	0.937	0.01	No
Gene duplicates retained from the α WGD	-8.505e-02	1.574e-02	5.402	$1*10^{-7}$	1.00	Yes
Gene duplicates retained from the βγ WGD	-1.225e-04	2.657e-03	0.046	0.963	0.01	No
Tandem duplicated genes	6.005e-05	2.679e-03	0.022	0.982	0.01	No

Genetic features	Estimated transcriptional noise F´					
	Coefficients	Adjusted Standard error	Z value	Pr(>|z|)	Relative variable importance	Included in best model
Log (mean expression level)	-0.2758927	0.0097403	28.33	$< 2*10^{-16}$	1.00	Yes
Log (gene length)	-0.1478755	0.0117618	12.57	$< 2*10^{-16}$	1.00	Yes
gbM	-0.0083983	0.0224336	0.374	0.708	0.15	No
Lethal gene	-0.0027356	0.0128541	0.213	0.831	0.06	No
Gene duplicates retained from the α WGD	-0.1148445	0.0212088	5.415	$1*10^{-7}$	1.00	Yes
Gene duplicates retained from the βγ WGD	-0.0002369	0.0039510	0.060	0.952	0.01	No
Tandem duplicated genes	0.0005671	0.0061605	0.092	0.927	0.02	No

Table S5. Linear regression model including gbM and a set of orthogonal predictor variables generated by a PCR analyses including gene length, gene expression breadth and gene duplicates retained from the α WGD as well as mean expression level in the case of F' to predict gene expression noise (F* and F'). The loadings of each genomic feature on the orthogonal predictor variables are shown in the supplementary Fig. S2 and S3.

**Predictors**	**Estimated stochastic gene expression F***			**Predictors**	**Estimated transcriptional noise F´**		
	Estimate	Standard Error	*p*-value		Estimate	Standard Error	*p*-value
Intercept	1.199849	0.013678	$< 2*10^{-16}$	Intercept	3.07124	0.01817	$< 2*10^{-16}$
PC 1	-0.077306	0.010311	$7.52*10^{-14}$	PC 1	0.11887	0.01292	$< 2*10^{-16}$
PC 2	0.072764	0.009221	$3.58*10^{-15}$	PC 2	-0.23562	0.01245	$< 2*10^{-16}$
PC 3	0.033624	0.010533	0.00142	PC 3	0.02942	0.01283	0.0219
				PC 4	-0.15561	0.01418	$< 2*10^{-16}$
gbM	-0.007193	0.023379	0.75834	gbM	-0.02715	0.03108	0.3824

Table S6. Model averaged ANCOVA of F* as well as F' with all genomic features studied using only genes identified as gbM or unmethylated in the two independent studies, Bewick et al. 2016 and Seymour et al. 2014 (R^2=0.0175 and R^2=0.0909, respectively; n=5 064). Genomic features which were kept in the best model generated by a BIC model averaging model selection are indicated in the last column.

**Genetic features**	**Estimated stochastic gene expression F***					
	Coefficients	Adjusted Standard error	Z value	Pr(>|z|)	Relative variable importance	Included in best model
Mean expression level	-2.148e-04	2.031e-03	0.106	0.9158	0.02	No
Log (gene length)	-7.832e-05	9.820e-03	7.976	< $2*10^{-16}$	1.00	Yes
gbM	-7.830e-05	3.085e-03	0.025	0.9798	0.01	No
Lethal gene	6.499e-04	5.956e-03	0.109	0.9131	0.02	No
A. lyrata homolog *Ka/Ks*	4.465e-04	3.009e-03	0.148	0.8820	0.03	No
Co-expression module size	-9.752e-05	1.506e-03	0.065	0.9484	0.02	No
Expression breadth	-6.580e-02	9.745e-03	6.752	< $2*10^{-16}$	1.00	Yes
Gene duplicates retained from the α WGD	-5.682e-02	3.418e-02	1.663	0.0964	0.80	Yes
Gene duplicates retained from the βγ WGD	4.637e-05	3.541e-03	0.013	0.9896	0.01	No
Tandem duplicated genes	1.118e-04	4.557e-03	0.025	0.9804	0.01	No

**Genetic features**	**Estimated transcriptional noise F´**					
	Coefficients	Adjusted Standard error	Z value	Pr(>|z|)	Relative variable importance	Included in best model
Log (mean expression level)	-0.2550540	0.0131291	19.43	< $2*10^{-16}$	1.00	Yes
Log (gene length)	-0.1148675	0.0132932	8.641	< $2*10^{-16}$	1.00	Yes
gbM	-0.0005852	0.0062898	0.093	0.9259	0.02	No
Lethal gene	0.0003096	0.0052965	0.058	0.9534	0.02	No
A. lyrata homolog *Ka/Ks*	0.0020687	0.0079220	0.261	0.7940	0.08	No
Co-expression module size	-0.0018415	0.0074879	0.246	0.8057	0.07	No
Expression breadth	-0.1226301	0.0130710	9.382	< $2*10^{-16}$	1.00	Yes
Gene duplicates retained from the α WGD	-0.0882174	0.0414618	2.128	0.0334	0.88	Yes
Gene duplicates retained from the βγ WGD	0.0003340	0.0056098	0.060	0.9525	0.02	No
Tandem duplicated genes	0.0002254	0.0062822	0.036	0.9714	0.01	No

Table S7. Model averaged ANCOVA of gene expression consistency (R^2=0.546; n=5 637). Genomic features which were kept in the best model generated by a BIC model averaging model selection are indicated in the last column.

Genomic features	Gene expression consistency					
	Coefficients	Adjusted Standard error	Z value	Pr(>|z|)	Relative variable importance	Included in best model
Log (mean expression level)	4.219	5.384e-02	78.364	$< 2*10^{-16}$	1.00	Yes
Log (gene length)	1.406	8.233e-02	17.074	$< 2*10^{-16}$	1.00	Yes
gbM	2.946e-01	2.336e-01	1.261	0.207	0.67	Yes
Lethal gene	-1.802e-03	2.402e-02	0.075	0.940	0.02	No
A. lyrata homolog Ka/Ks	-3.349e-02	6.519e-02	0.514	0.607	0.24	No
Co-expression module size	7.287e-02	8.960e-02	0.813	0.416	0.45	No
Expression breadth	5.269e-01	5.509e-02	9.566	$< 2*10^{-16}$	1.00	Yes
Gene duplicates retained from the α WGD	5.190e-01	1.180e-01	4.398	$1.1*10^{-5}$	1.00	Yes
Gene duplicates retained from the $\beta\gamma$ WGD	-6.796e-05	1.847e-02	0.004	0.997	0.01	No
Tandem duplicated genes	1.589e-03	2.823e-02	0.056	0.955	0.02	No

Table S8. Linear regression model including gbM and a set of orthogonal predictor variables generated by a PCR analyses including mean expression level, gene length, gene expression breadth and gene duplicates retained from the α WGD to predict gene expression consistency. The loadings of each genomic feature on the orthogonal predictor variables are shown in the supplementary Fig. S4.

**Predictors**	**Log (intron FPKM)**		
	Estimate	Standard Error	*p*-value
Intercept	12.06959	0.07807	$< 2*10^{-16}$
PC 1	-1.96386	0.05552	$< 2*10^{-16}$
PC 2	2.55328	0.05352	$< 2*10^{-16}$
PC 3	0.34468	0.05516	$4.43*10^{-10}$
PC 4	3.09063	0.06095	$< 2*10^{-16}$
gbM	0.45663	0.13357	0.000633

Table S9. Model averaged ANCOVA of gene expression consistency using only genes identified as gbM or unmethylated in the two independent studies, Bewick et al. 2016 and Seymour et al. 2014 (R^2=0.549; n=5 064). Genomic features which were kept in the best model generated by a BIC model averaging model selection are indicated in the last column.

**Genomic features**	**Gene expression consistency**					
	Coefficients	Adjusted Standard error	Z value	Pr(>|z|)	Relative variable importance	Included in best model
Log (mean expression level)	4.2545812	0.0562651	75.617	$< 2*10^{-16}$	1.00	Yes
Log (gene length)	1.3531769	0.0798001	16.957	$< 2*10^{-16}$	1.00	Yes
gbM	0.5052635	0.1940094	2.604	0.00921	0.93	Yes
Lethal gene	-0.0033970	0.0325469	0.104	0.91687	0.02	No
A. lyrata homolog Ka/Ks	-0.0153532	0.0453320	0.339	0.73485	0.13	No
Co-expression module size	0.0169273	0.0488635	0.346	0.72903	0.13	No
Expression breadth	0.5375005	0.0567918	9.464	$< 2*10^{-16}$	1.00	Yes
Gene duplicates retained from the α WGD	0.4375515	0.1524944	2.869	0.00411	0.95	Yes
Gene duplicates retained from the $\beta\gamma$ WGD	-0.0021089	0.0272180	0.077	0.93824	0.02	No
Tandem duplicated genes	0.0001359	0.0251099	0.005	0.99568	0.01	No

Table S10. Model averaged ANCOVA of log (intron FPKM) and various genomic features (R^2=0.728; n=4 376). Genomic features which were kept in the best model generated by a BIC model averaging model selection are indicated in the last column.

Genomic features	Log (intron FPKM)					
	Coefficients	Adjusted Standard error	Z value	Pr(>|z|)	Relative variable importance	Included in best model
Log (gene FPKM)	1.560	1.584e-02	98.488	$< 2*10^{-16}$	1.00	Yes
Log (gene length)	-8.398e-01	3.151e-02	26.650	$< 2*10^{-16}$	1.00	Yes
Log (total intron length)	4.618e-01	2.929e-02	15.767	$< 2*10^{-16}$	1.00	Yes
Log (intron number)	8.906e-01	2.901e-02	30.705	$< 2*10^{-16}$	1.00	Yes
gbM	-1.107e-01	6.132e-02	1.806	0.071	0.83	Yes
Lethal gene	6.777e-04	7.316e-03	0.093	0.926	0.02	No
A. lyrata homolog Ka/Ks	4.367e-04	3.683e-03	0.119	0.906	0.03	No
Co-expression module size	1.598e-04	2.491e-03	0.064	0.949	0.02	No
Expression breadth	-8.594e-02	1.606e-02	5.350	$1*10^{-7}$	1.00	Yes
Gene duplicates retained from the α WGD	-4.469e-04	5.608e-03	0.080	0.936	0.02	No
Gene duplicates retained from the $\beta\gamma$ WGD	9.661e-05	5.813e-03	0.017	0.987	0.02	No
Tandem duplicated genes	-3.749e-04	8.088e-03	0.046	0.963	0.02	No

Table S11. Linear regression model including gbM and a set of orthogonal predictor variables generated by a PCR analyses including gene FPKM, total intron length, number of introns, gene length and expression breadth to predict the number of reads mapping to the introns of a gene. The loadings of each genomic feature on the orthogonal predictor variables are shown in the supplementary Fig. S5.

**Predictors**	**Log (intron FPKM)**		
	Estimate	Standard Error	*p*-value
Intercept	2.323141	0.024263	$< 2*10^{-16}$
PC 1	0.001077	0.011511	0.925
PC 2	-0.959228	0.014663	$< 2*10^{-16}$
PC 3	-1.303276	0.016856	$< 2*10^{-16}$
PC 4	1.198321	0.031490	$< 2*10^{-16}$
PC 5	-0.294199	0.036532	$1.03*10^{-16}$
gbM	-0.133017	0.039130	$6.81*10^{-04}$

Table S12. Model averaged ANCOVA of log (intron FPKM) using only genes identified as gbM or unmethylated in the two independent studies, Bewick et al. 2016 and Seymour et al. 2014 (R^2=0.726; n=3 958). Genomic features which were kept in the best model generated by a BIC model averaging model selection are indicated in the last column**.**

**Genomic features**	**Log (intron FPKM)**					
	Coefficients	Adjusted Standard error	Z value	Pr(>|z|)	Relative variable importance	Included in best model
Log (gene FPKM)	1.5734218	0.0168737	93.247	$< 2*10^{-16}$	1.00	Yes
Log (gene length)	-0.8402360	0.0318756	26.360	$< 2*10^{-16}$	1.00	Yes
Log (total intron length)	0.4789313	0.0311087	15.395	$< 2*10^{-16}$	1.00	Yes
Log (intron number)	0.8957627	0.0308620	29.025	$< 2*10^{-16}$	1.00	Yes
gbM	-0.1538826	0.0536911	2.866	0.00416	0.95	Yes
Lethal gene	0.0007564	0.0079670	0.095	0.92436	0.02	No
A. lyrata homolog Ka/Ks	0.0004346	0.0037858	0.115	0.90860	0.03	No
Co-expression module size	0.0006164	0.0046021	0.134	0.89345	0.03	No
Expression breadth	-0.0828208	0.0169416	4.889	$1*10^{-6}$	1.00	Yes
Gene duplicates retained from the α WGD	-0.0005220	0.0062212	0.084	0.93313	0.02	No
Gene duplicates retained from the $\beta\gamma$ WGD	0.0000738	0.0062544	0.012	0.99058	0.02	No
Tandem duplicated genes	-0.0008410	0.0105740	0.080	0.93661	0.02	No

Table S13. Best model to predict gbM based on a BIC model selection.

**Predictors**	**gbM**		
	Estimate	Standard Error	*p*-value
Intercept	0.483415	0.006029	$< 2*10^{-16}$
Log (total intron length)	-0.040660	0.009399	$1.55*10^{-05}$
Log (gene length)	0.312428	0.009435	$< 2*10^{-16}$
Co-expression module size	0.073251	0.006234	$< 2*10^{-16}$
Expression breadth	0.056868	0.006120	$< 2*10^{-16}$
Log (mean expression level)	-0.018734	0.006177	0.00244

Supplementary Figures

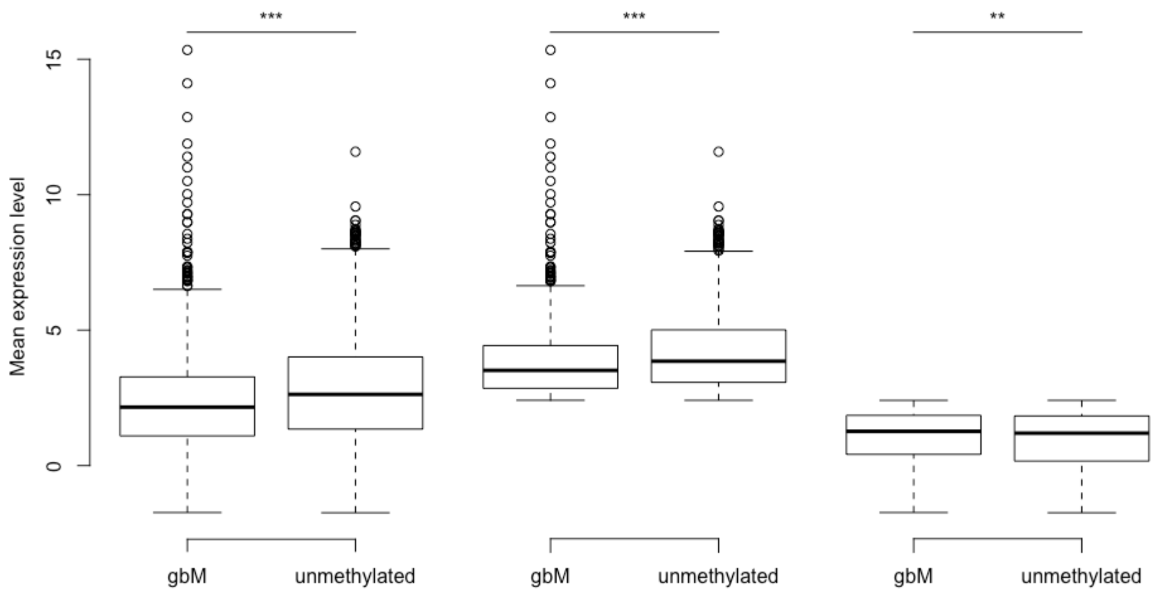

Figure S1. Differences in the mean expression level of gbM and unmethylated genes. On the left, the distribution of the expression level of all genes split into gbM and unmethylated genes. In the middle, the distribution of the expression level of genes with an expression level higher that the median split into gbM and unmethylated genes. On the right, the distribution of the expression level of genes with an expression level lower that the median split into gbM and unmethylated genes. Significances were based on a two-sided Mann-Whitney U-test.

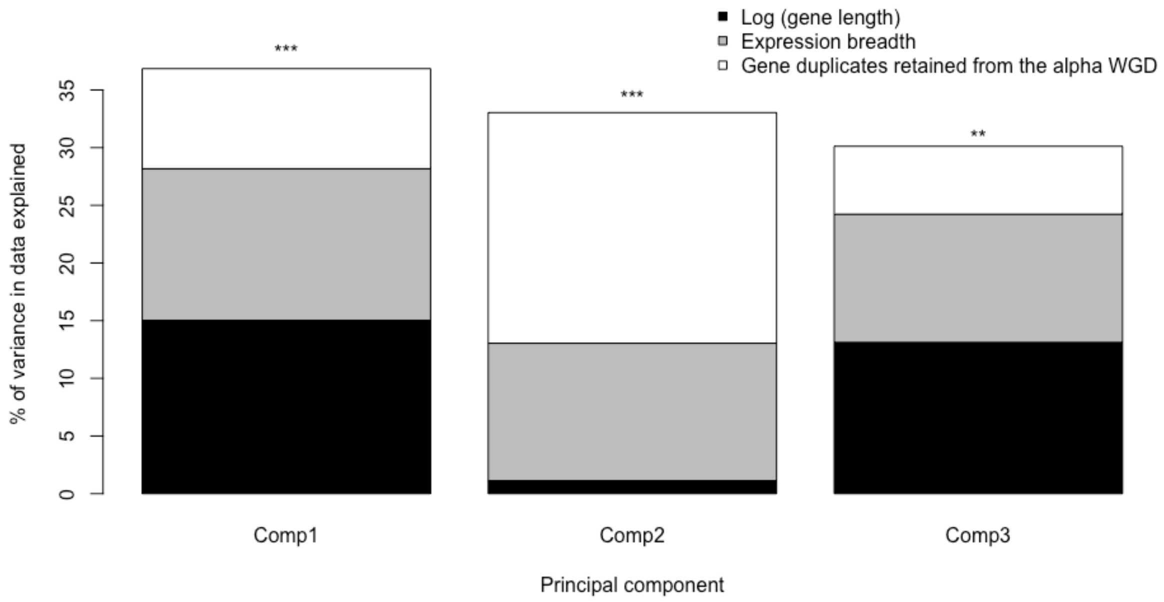

Figure S2. Loadings of the different genomic features on each principal component used in the linear regression analyses including gbM and this set of orthogonal predictor variables to predict F*. Principal components which were significantly correlated with F* are indicated with stars (**: *p*-value < 0.01; ***: *p*-value < 0.001).

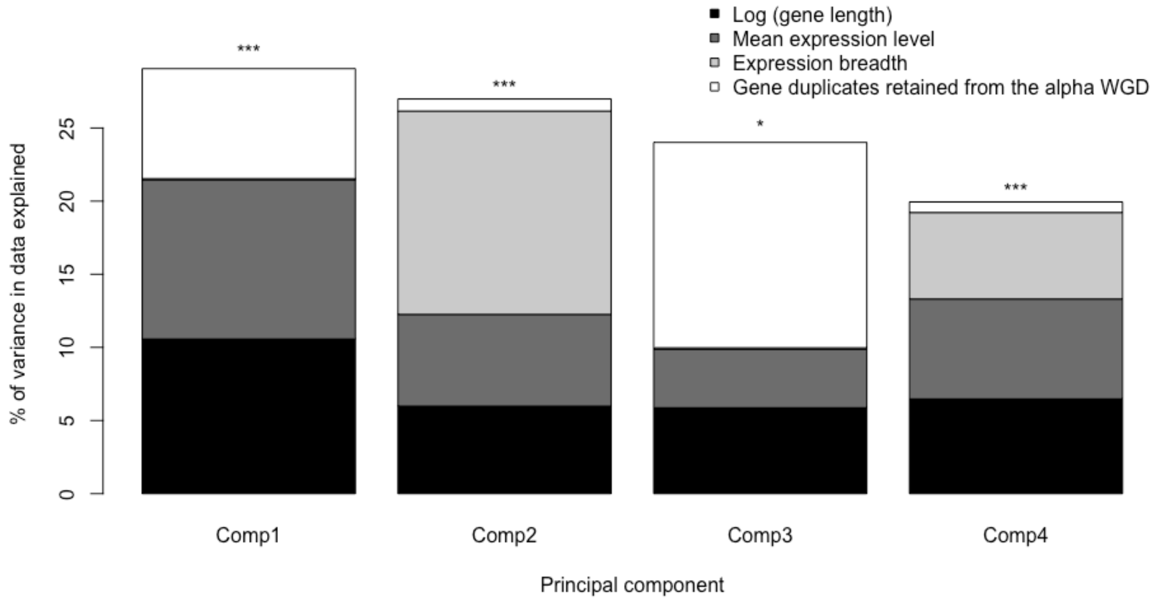

Figure S3. Loadings of the different genomic features on each principal component used in the linear regression analyses including gbM and this set of orthogonal predictor variables to predict F'. Principal components which were significantly correlated with F' are indicated with stars (*: *p*-value < 0.05; ***: *p*-value < 0.001).

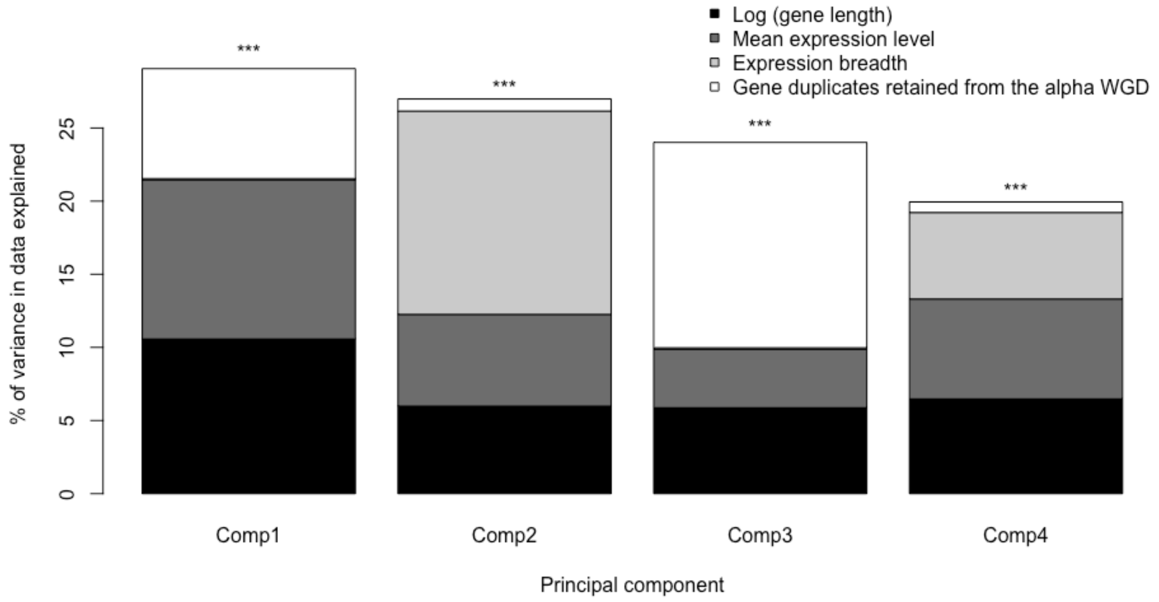

Figure S4. Loadings of the different genomic features on each principal component used in the linear regression analyses including gbM and this set of orthogonal predictor variables to predict gene expression consistency. Principal components which were significantly correlated with gene expression consistency are indicated with stars (***: *p*-value < 0.001).

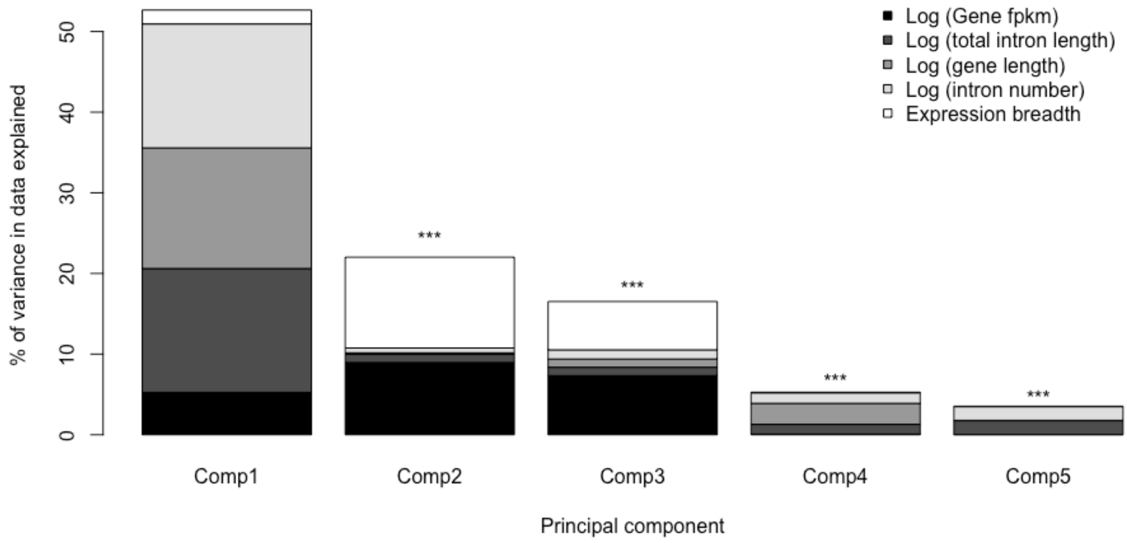

Figure S5. Loadings of the different genomic features on each principal component used in the linear regression analyses including gbM and this set of orthogonal predictor variables to predict the number of reads mapping to the introns of a gene. Principal components which were significantly correlated with the number of reads mapping to the introns of a gene are indicated with stars (***: *p*-value < 0.001).
